# Correlation between LCX-QFR and clinical outcomes following a single-stent strategy for left main bifurcation lesions

**DOI:** 10.3389/fcvm.2025.1578159

**Published:** 2025-05-30

**Authors:** Cheng-Duo Zhang, Ji-Sheng Zhou, Li-Yun He, Xin-Ye Xu, Yu-Peng Wang, Ming Cui

**Affiliations:** Department of Cardiology, NHC Key Laboratory of Cardiovascular Molecular Biology and Regulatory Peptides, Peking University Third Hospital, Beijing, China

**Keywords:** left main coronary artery disease, quantitative flow ratio fraction, percutaneous coronary intervention, main adverse cardiac events, left main bifurcation lesions

## Abstract

**Objective:**

The aim of this study was to investigate the quantitative flow ratio (QFR) outcomes in the left circumflex artery (LCX) following the placement of a crossover stent from the left main coronary artery (LM) to the left anterior descending artery (LAD) in LM bifurcation lesions. In addition, we sought to assess the relationship between these QFR results and clinical prognoses.

**Background:**

The treatment approach for LM bifurcation lesions remains a topic of debate, with the LM-LAD single-stent technique being one possible option. QFR, a fractional flow reserve calculation method derived from angiography that does not require pressure guide wires, could serve as an alternative functional assessment of the LCX. This study aims to evaluate the clinical outcomes of postoperative LCX by utilizing QFR measurements, addressing a current gap in the relevant literature on this topic.

**Methods:**

This study was a retrospective, single-center analysis of patients with LM bifurcation lesions who underwent percutaneous coronary intervention (PCI) guided by intravascular ultrasound. QFR values were derived from angiographies. The primary endpoint was the 1-year rate of major adverse cardiac events, defined as a composite of cardiovascular death, target bifurcation-related myocardial infarction (MI), or target bifurcation revascularization. The secondary clinical endpoint was defined as the persistence or recurrence of angina pectoris after PCI.

**Results:**

We analyzed 91 patients from a total of 180 who were screened for LM bifurcation lesions. All patients completed the 1-year follow-up. The pre- and post-PCI QFR values were 0.89 ± 0.09 and 0.86 ± 0.11, respectively. Subgroup analysis showed that 74 patients were in the postoperative QFR ≥0.80 group, whereas 17 patients were in the QFR <0.80 group. In addition, 32 patients had a ΔQFR ≥0, and 58 patients had a ΔQFR <0. Nine patients (9.9%) achieved the primary endpoint, including one patient with non-ST elevation myocardial infarction who received revascularization in both the LM-LAD and LCX arteries. In addition, nine patients (9.9%) reported no substantial improvement in their chest pain symptoms. Post-LCX-QFR <0.8 was associated with a higher 1-year incidence of cardiovascular death or MI (*P* = 0.036). ΔQFR proved to be a robust predictor of the 1-year incidence of the primary endpoint, with an incidence of 15.3% in the ΔQFR ≥0 group compared to 0% in the ΔQFR <0 group (area under the curve: 0.822; 95% CI: 0.728–0.895, *P* < 0.001), especially when ΔQFR ≤−0.03.

**Conclusions:**

After the LM-LAD single-stent strategy for LM bifurcation lesions, a ΔQFR of LCX ≤−0.03 was associated with a higher risk of 1-year main adverse cardiac events, indicating the superior prognostic value of the post-PCI physiological assessment.

## 1 Introduction

Coronary artery bifurcation lesions (CBLs) are common and carry a higher risk of main cardiac events and restenosis after percutaneous coronary intervention (PCI) compared to lesions alone (1). The optimal treatment strategy for coronary bifurcation anatomy remains a subject of debate. CBLs with extensive atherosclerosis involving a large and significantly diseased side branch (SB) may benefit from an elective two-stent bifurcation technique (2). However, trials involving bifurcated lesions have shown that a systematic dual drug-eluting stent (DES) strategy offers no advantage, and in fact, a more sophisticated approach may lead to more severe long-term mortality (3). A provisional single-stent strategy may be superior to the two-stent method and is considered a feasible treatment option for left main coronary artery (LM) bifurcation lesions, although there are significant differences in the predictors, functional significance, and luminal changes for compromised SB after main vessel dilation (4, 5).

According to the “provisional” strategy, SB intervention should be considered only when the SB result is suboptimal. However, a suboptimal result for the left circumflex artery (LCX) ostium has not been standardized. The utilization of fractional flow reserve (FFR) to guide interventions in the LCX has been proposed as a means to enhance clinical outcomes by reducing unnecessary side branch interventions. However, it should be noted that FFR-guided LCX intervention may pose technical challenges, such as difficulty accessing side branches through stent struts, and its superiority over angiography-guided LCX intervention remains unclear (6, 7).

Therefore, we conducted a comprehensive analysis of the alterations in LCX-quantitative flow ratio (QFR) values following intravascular ultrasound (IVUS)-guided LM to left anterior descending artery (LAD) crossover stenting, and explored their correlation with clinical outcomes.

## 2 Methods

### 2.1 Population

This was a single-center, retrospective, observational study of patients with significant *de novo* LM stem coronary artery disease, defined as angiographic stenosis >50%, who presented with distal bifurcation lesions classified as Medina (1,1,0/1,1,1) and had mild-to-moderate LCX disease with angiographic stenosis <70%. All the DESs were implanted from the LM to the LAD. The study was conducted at Peking University Third Hospital between January 2019 and December 2023. Adults (≥18 years old) who were planning to undergo PCI treatment based on coronary angiography (CAG) findings were eligible for inclusion.

The main exclusion criteria encompassed: (1) use of a systematic dual-stent strategy or provisional intervention in the LCX ostium, (2) presence of acute myocardial infarction (MI) or severe left ventricular dysfunction, (3) history of bypassed vessels, (4) diffuse LCX disease extending >7 mm from the LCX ostium, (5) an LCX-angiographic diameter <2.5 mm, and (6) other conditions that prevented post-PCI QFR analysis.

### 2.2 PCI procedure

All patients received a loading dose of antiplatelet treatment before the procedure. All patients underwent DES implantation from the LM to the LAD via a simple crossover intervention technique, with stent diameter and length selected by expert operators based on the reference vessel diameter, culprit lesion length, plaque burden (PB), and partly IVUS-based measurements. During PCI, the type of DES employed, stenting techniques, utilization of intravascular ultrasound, and application of poststent adjunctive balloon inflation were left to the discretion of the operating physicians. In cases where the LCX flow restriction occurred during LM-to-LAD stent implantation, the surgeon used balloon dilation to improve it.

All patients received standard dual antiplatelet therapy and other secondary prevention drugs for coronary heart disease after discharge. During follow-up, drugs and dosages were adjusted according to each patient's blood pressure, heart rate, clinical symptoms, comorbidities, and the operator’s discretion.

### 2.3 QFR and rQFR analysis

A certified operator, qualified to conduct QFR analysis, performed all measurements using QFR software [AngioPlus 2.0.1.0 and PStation (Pulse Medical Technology, Shanghai, China)], while a second, more experienced operator reviewed and made corrections to all analyses as needed. The results provided by the second operator were utilized for the analyses.

**Figure 1:**
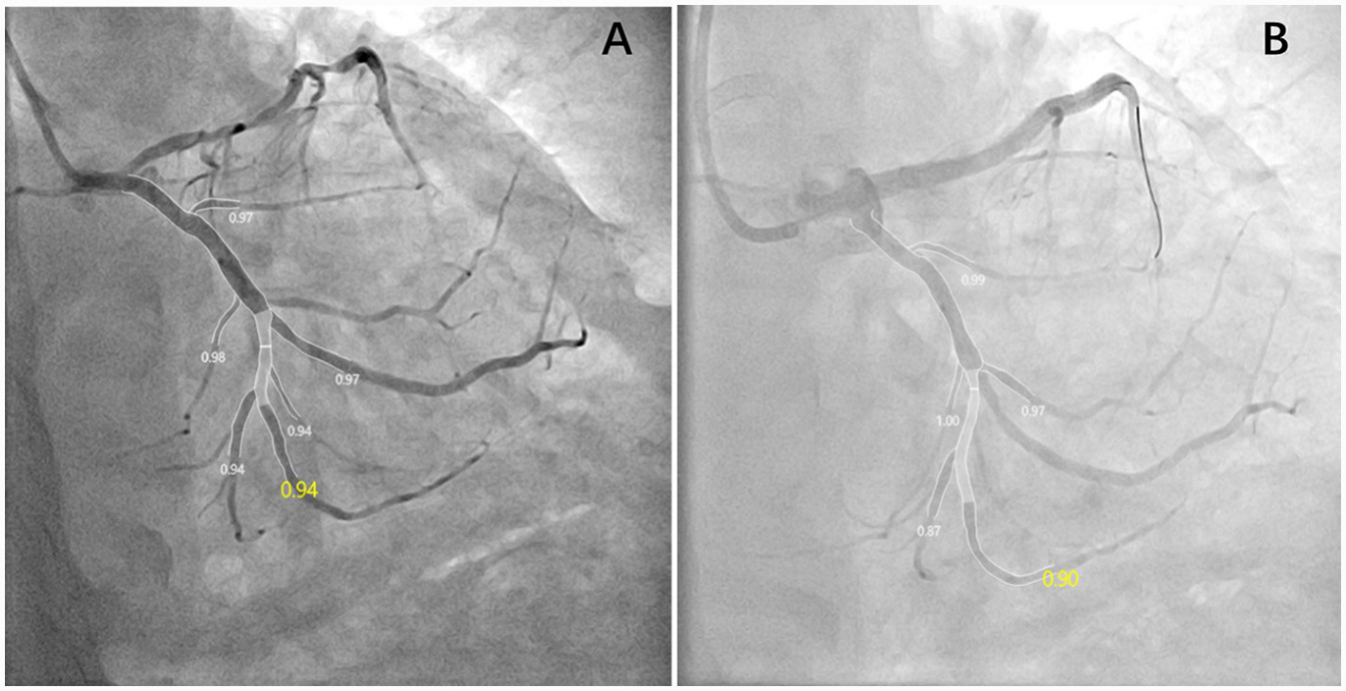
Key frames of the appropriate angiographic views for QFR assessment pre- **(A)** and post-PCI **(B)**. This patient suffered a decline in post-PCI QFR, and the ΔQFR was −0.04.

Eligible patients undergoing LM-LAD stenting were enrolled. Angiographic images with optimal contrast opacification and clear visualization of all coronary artery branches, including the ostium of the LCX, were selected. QFR analysis was performed in a blinded fashion. Independent operators, blinded to each other's analyses, selected appropriate angiographic views and keyframes for QFR assessment to minimize interobserver variability. This approach ensured an objective and unbiased assessment of LCX ostial stenosis (Figure 1). QFR measurements, including hemodynamic parameters and FFR-derived indices, were acquired. These parameters included blood flow velocity, microvascular resistance, QFR, resting QFR (rQFR), and the difference between pre- and post-PCI QFR (ΔQFR). These parameters were employed to evaluate the impact of LM-LAD stenting on LCX hemodynamics.

### 2.4 IVUS imaging

IVUS was performed either pre- and post-PCI or only postoperatively, according to the surgeon's experience with LM-LAD stent implantation. After the appropriate selection of a guiding catheter based on the target vessel's lesion characteristics, the IVUS catheter was advanced distal to the lesion, and a pullback was performed to acquire intravascular images. In conjunction with CAG findings, quantitative IVUS analysis was performed on the proximal and distal segments of the LM, LAD, and partly LCX arteries. The measured parameters included lumen diameter, lumen area, plaque burden, and lesion length. Poststenting IVUS was performed within the LM-LAD segment to evaluate stent apposition, stent expansion (minimum stent area), and lumen dimensions (diameter and area). The decision to perform an IVUS assessment of the LCX ostium was made based on intraoperative findings and clinical judgment.

### 2.5 Clinical outcomes

The primary endpoint was the 1-year rate of major adverse cardiac events (MACE), which was defined as a composite of cardiovascular death, target bifurcation-related MI, or target bifurcation revascularization. All deaths were assumed to be of cardiac origin unless a clear non-cardiac cause was reported. Target bifurcation-related MI was defined as any MI with angiographic evidence confirming that the culprit lesion corresponded to the previously treated target bifurcation. Target bifurcation revascularization was defined as repeat percutaneous intervention or bypass surgery for culprit target lesions exhibiting restenosis or occlusion at the LCX ostium or within 5 mm of the distal or proximal edge of the implanted stent. The secondary clinical endpoint was persistent or recurrent angina pectoris after PCI. Clinical follow-up was performed at 2, 4, 6, 8, 10, and 12 months via outpatient clinical visits or telephone interviews.

### 2.6 Statistical analysis

Data were processed using SPSS 21.0 (SPSS, Inc., an IBM Company), GraphPad Prism 8.0.0 (GraphPad Software, San Diego, CA, USA), MedCalc 22.014 (MedCalc Software Ltd., Belgium), and STATA 18.0 (StataCorp, USA). Intergroup comparisons for normally distributed data were made using *t*-tests or one-way ANOVA. The counting data rate (%) or constituent ratio was expressed, and Pearson's chi-square test was used for comparison between groups. A receiver operating characteristic (ROC) curve analysis was performed to determine the optimal cutoff values for parameters that independently forecast ΔQFR following stenting in the LCX. The Kaplan–Meier method was employed to determine the time to clinical endpoints and survival rates. A forest plot was generated to display independent predictors of postoperative QFR. All *P*-values were two-tailed, and *P* < 0.05 was considered statistically significant.

## 3 Results

### 3.1 Study population

**Figure 2:**
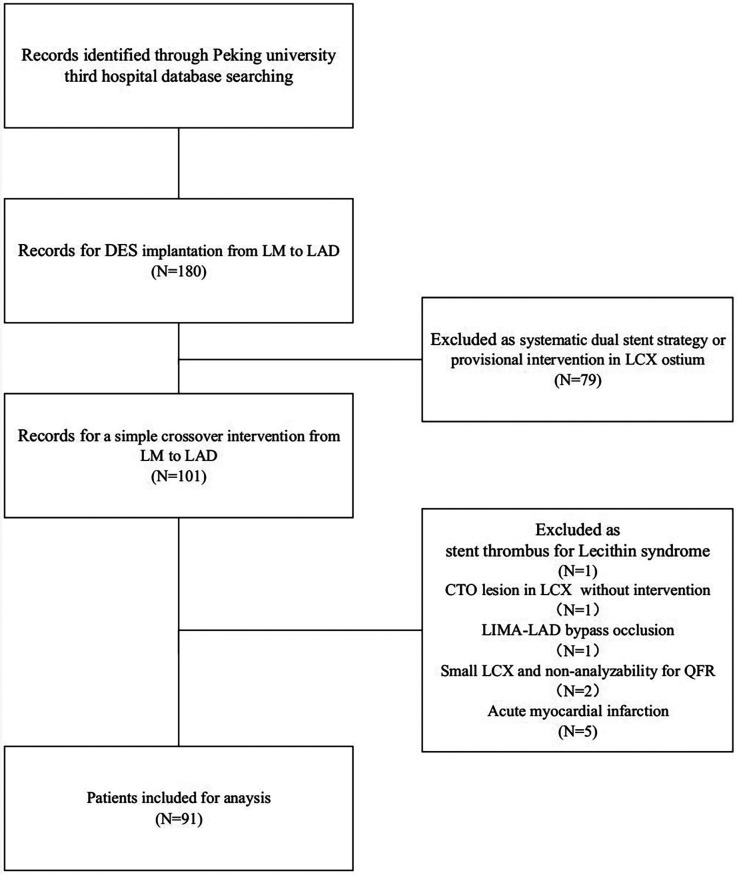
Flowchart of patient selection.

A comprehensive review of patients who underwent LM-LAD DES implantation at Peking University Third Hospital between 2019 and 2024 identified a total of 180 patients. Among them, 79 patients were treated with a dual-stent strategy or underwent provisional intervention at the LCX ostium and were therefore excluded. This left 101 patients who received a straightforward crossover intervention from the LM to LAD. Ultimately, 91 patients met the inclusion criteria (refer to Figure 2). Of these 91 patients, IVUS was utilized both preoperatively and postoperatively in 53 patients; specifically, 5 patients underwent pre-PCI IVUS evaluation to guide treatment strategy, while 33 patients received IVUS assessment conducted exclusively during postdilatation. Notably, only 14 patients had preoperative IVUS measurements taken from the LCX to the LM.

The pre-PCI LCX-QFR was 0.89 ± 0.09 among the 91 patients. Following PCI, a subgroup analysis was performed to evaluate whether the LCX-QFR exceeded 0.80 and to examine changes in the LCX-QFR values. This analysis revealed that 74 patients fell into the postoperative QFR ≥0.80 category, while 17 patients were classified in the QFR <0.80 group. In addition, 32 patients had a ΔQFR ≥0, and 58 patients had a ΔQFR <0. The baseline clinical, angiographic, procedural, and quantitative coronary angiography (QCA) characteristics of the patients in these subgroups are detailed in Tables 1, 2. A comparison indicated that patients with a ΔQFR <0 had a higher prevalence of diabetes mellitus (*P* = 0.042) and a greater incidence of stable coronary artery disease (SCAD) (*P* = 0.045). Furthermore, patients in the postoperative QFR ≥0.80 subgroup exhibited elevated pre-PCI QFR levels (*P* < 0.01) and a larger LM area following stenting (*P* = 0.016).

**Table 1:** Clinical characteristics of patients with post-QFR ≥0.80 or <0.80 and ΔQFR ≥0 or <0.

Variables	Total (*N* = 91 patients)	Post-QFR ≥ 0.80 (*N* = 74 patients)	Post-QFR<0.80 (*N* = 17 patients)	*P*-value	ΔQFR ≥ 0 (*N* = 32 patients)	ΔQFR<0 (*N* = 59 patients)	*P*-value
Age (years)	68 ± 9	67 ± 9	70 ± 9	0.389	67 ± 9	69 ± 9	0.340
Male patients, *n* (%)	67 (73.6)	54 (73.0)	13 (76.5)	0.516	22 (68.8)	45 (76.3)	0.296
Cardiovascular risk factors							
Hypertension, *n* (%)	68 (74.7)	53 (71.6)	15 (88.2)	0.170	22 (68.8)	46 (78.0)	0.265
Diabetes mellitus, *n* (%)	33 (36.3)	26 (35.1)	7 (41.2)	0.450	7 (21.9)	26 (44.1)	0.045*
Hypercholesterolemia, *n* (%)	39 (42.9)	33 (44.6)	6 (35.3)	0.384	14 (43.8)	25 (42.4)	0.500
Smoker, *n* (%)	44 (48.4)	38 (51.4)	6 (35.3)	0.407	17 (53.1)	27 (45.8)	0.283
Clinical presentation							
SCAD	7 (7.7)	5 (6.8)	2 (11.8)	0.389	0 (0)	7 (11.9)	0.042*
UAP	84 (92.3)	69 (93.2)	15 (88.2)	0.389	32 (100)	52 (88.1)	
Creatinine, μmol/L	83 ± 21	82 ± 21	86 ± 20	0.435	78 ± 17	85 ± 23	0.136
HbA1c (%)	6.5 ± 1.1	6.5 ± 1.1	6.8 ± 1.2	0.439	6.3 ± 0.8	6.7 ± 1.2	0.117
LDL-C (mmol/L)	2.12 ± 0.81	2.15 ± 0.81	2.02 ± 0.85	0.574	2.06 ± 0.89	2.20 ± 0.77	0.583
Left ventricular ejection fraction (%)	65 ± 10	66 ± 10	63 ± 12	0.234	66 ± 11	65 ± 9	0.444
Body mass index (kg m^2^)	22.9 ± 6.7	23.2 ± 6.33	21.8 ± 8.3	0.441	23.0 ± 6.5	22.3 ± 6.9	0.924

UAP, unstable angina pectoris; SCAD, stable coronary artery disease; LDL-C, low-density lipoprotein cholesterol.

*P < 0.05.

**Table 2:** Lesion and procedural characteristics of the study population.

Variables	Total (*N* = 91 patients)	Post-QFR ≥ 0.80 (*N* = 74 patients)	Post-QFR < 0.80 (*N* = 17 patients)	*P*-value	ΔQFR ≥ 0 (*N* = 32 patients)	ΔQFR < 0 (*N* = 59 patients)	*P*-value
Baseline							
LM							
MLA (mm^2^)	6.46 ± 3.74	6.63 ± 4.01	5.53 ± 1.58	0.424	5.43 ± 3.38	6.93 ± 3.84	0.162
MLD (mm)	2.38 ± 0.74	2.41 ± 0.79	2.20 ± 0.33	0.430	2.17 ± 0.68	2.48 ± 0.74	0.136
PB (%)	67 ± 12	66 ± 13	71 ± 6	0.278	71 ± 11	66 ± 13	0.147
LAD							
MLA (mm^2^)	3.86 ± 1.51	3.79 ± 1.37	4.24 ± 2.16	0.414	4.33 ± 1.67	3.66 ± 1.41	0.129
MLD (mm)	1.93 ± 0.37	1.91 ± 0.34	2.09 ± 0.51	0.188	2.05 ± 0.46	1.89 ± 0.32	0.148
PB (%)	72 ± 8	72 ± 8	71 ± 10	0.701	70 ± 6	72 ± 9	0.424
After LM-LAD stent implantation							
LM							
MLA (mm^2^)	11.4 ± 2.74	11.8 ± 2.84	9.97 ± 1.55	0.016*	11.7 ± 2.96	11.3 ± 2.62	0.459
MLD (mm)	3.54 ± 0.49	3.60 ± 0.52	3.30 ± 0.26	0.031*	3.57 ± 0.54	3.53 ± 0.47	0.730
LAD							
MLA, (mm^2^)	8.11 ± 2.04	8.21 ± 1.94	7.64 ± 2.49	0.315	7.98 ± 1.95	8.17 ± 2.11	0.689
MLD (mm)	3.02 ± 0.40	3.05 ± 0.37	2.87 ± 0.53	0.101	3.02 ± 0.39	3.01 ± 0.42	0.906
PCI procedural data							
LM-LAD stent diameter (mm)	3.61 ± 0.40	3.64 ± 0.39	3.53 ± 0.44	0.331	3.58 ± 0.42	3.63 ± 0.39	0.518
Postdilatation performed (%)	70 (76.9)	58 (78.4)	12 (70.6)	0.492	25 (78.1)	45 (76.3)	0.315
Postdilataion diameter (mm)	3.90 ± 0.41	3.89 ± 0.67	3.75 ± 0.38	0.442	3.93 ± 0.41	3.82 ± 0.72	0.493
LCX physiology assessment							
Pre-PCI LCX-QFR	0.89 ± 0.09	0.92 ± 0.05	0.77 ± 0.10	<0.01*	0.89 ± 0.06	0.89 ± 0.10	0.473
Post-PCI LCX-QFR	0.86 ± 0.11	0.90 ± 0.05	0.69 ± 0.10	<0.01*	0.90 ± 0.06	0.83 ± 0.12	<0.01*

*P < 0.05.

### 3.2 Clinical follow-up

**Figure 3:**
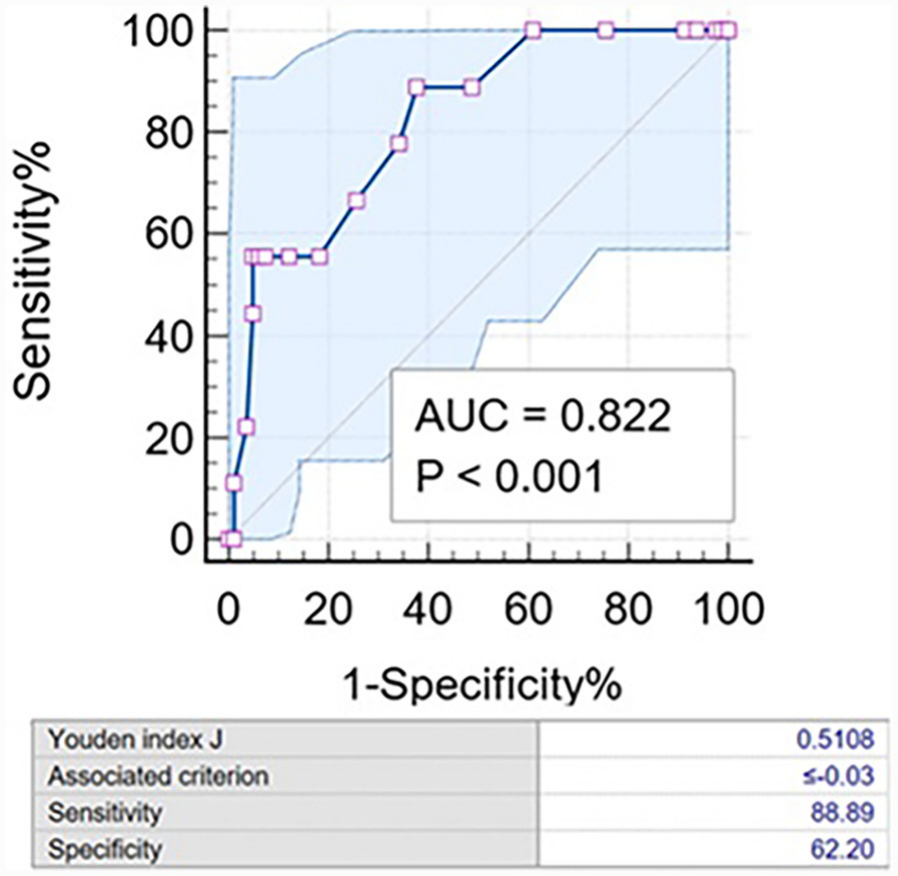
Characteristic curve of ΔQFR for the 1-year incidence of the primary endpoint. The AUC was 0.822 (95% CI: 0.694–0.951). The diagnostic sensitivity and specificity of the ΔQFR ≤−0.03 were 88.9% and 62.2%, respectively. AUC, area under the curve.

**Figure 4:**
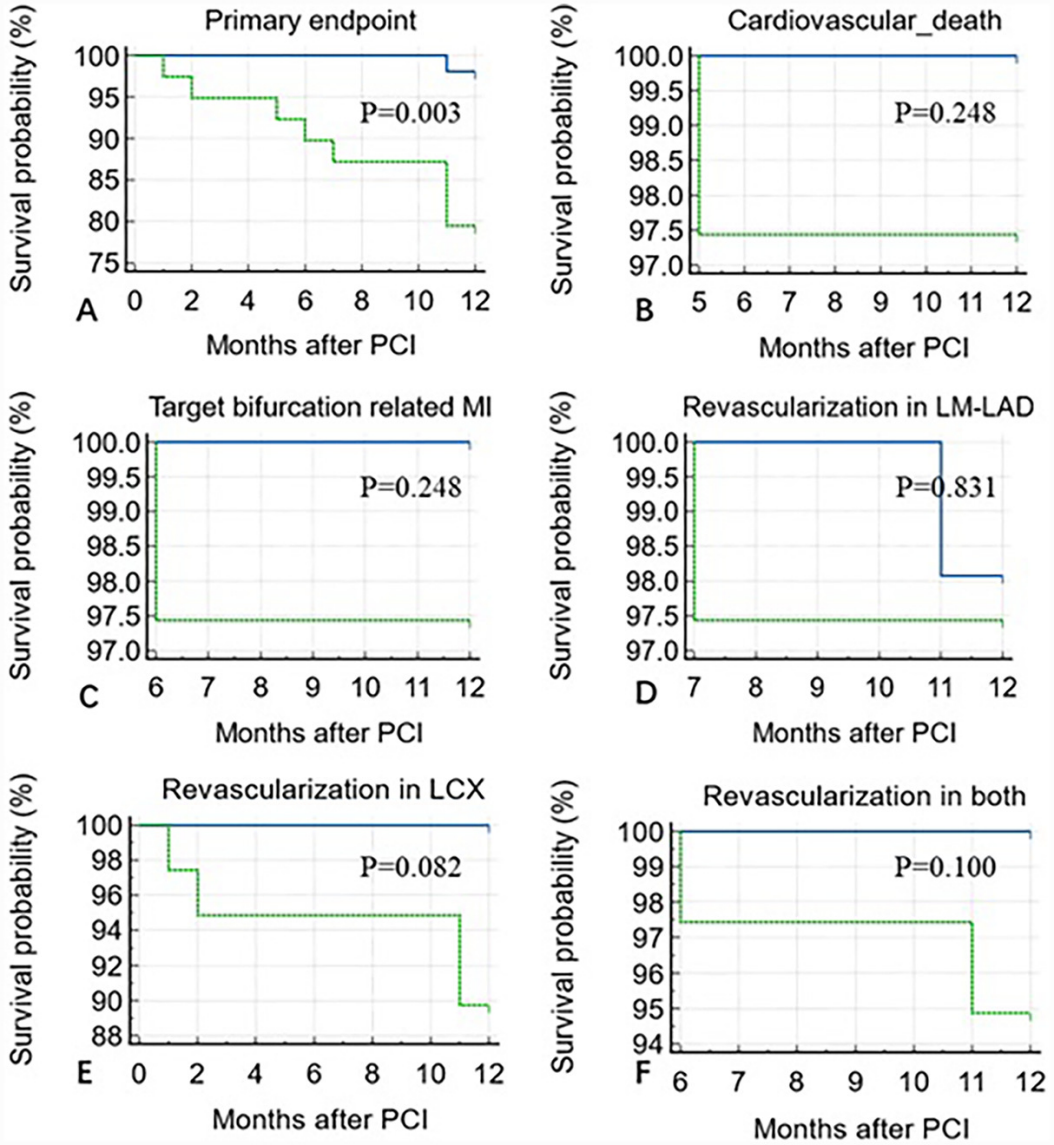
Kaplan–Meier analysis showing a higher incidence of 1-year primary endpoint in the ΔQFR <−0.03 group compared to the ΔQFR >−0.03 group (*P* = 0.003) over 12 months of follow-up after PCI **(A)**. No statistically significant differences were observed in cardiovascular death (*P* = 0.248) **(B)**, target bifurcation-related MI (*P* = 0.248) **(C)**, revascularization in LM-LAD (*P* = 0.831) **(D)**, LCX (*P* = 0.082) **(E)**, or both (*P* = 0.100) **(F)**.

Complete 1-year follow-up data were available for all 91 patients, and the results are presented in Table 3 and Figures 3, 4. Nine patients (9.9%) achieved the primary endpoint, which included one individual who experienced a non-ST elevation myocardial infarction (NSTEMI) outside of the hospital at 11 months. This patient subsequently underwent revascularization involving the LM-LAD and LCX arteries. In addition, nine patients (9.9%) who did not meet the primary endpoint during the 1-year follow-up reported no substantial improvement in their chest pain symptoms and were prescribed antianginal medication in an outpatient setting.

**Table 3:** Clinical outcomes for the study population at 1 year.

Variables	Total (*N* = 91 patients)	Post-QFR ≥ 0.80 (*N* = 74 patients)	Post-QFR<0.80 (*N* = 17 patients)	*P*-value	ΔQFR ≥ 0 (*N* = 32 patients)	ΔQFR < 0 (*N* = 59 patients)	*P*-value
Primary endpoint	9 (9.9%)	6 (8.1%)	3 (17.6%)	0.235	0 (0.0%)	9 (15.3%)	0.020*
Cardiovascular death	1 (1.1%)	0 (0.0%)	1 (5.9%)	0.036*	0 (0.0%)	1 (1.7%)	0.459
Target bifurcation-related MI	1 (1.1%)	0 (0.0%)	1 (5.9%)	0.036*	0 (0.0%)	1 (1.7%)	0.459
Target bifurcation revascularization in LM-LAD	2 (2.2%)	2 (2.7%)	0 (0.0%)	0.493	0 (0.0%)	2 (3.4%)	0.292
Target bifurcation revascularization in LCX	4 (4.4%)	3 (4.1%)	1 (5.9%)	0.740	0 (0.0%)	4 (6.8%)	0.132
Target bifurcation revascularization both in LM-LAD and LCX	2 (2.2%)	1 (1.4%)	1 (5.9%)	0.251	0 (0.0%)	2 (3.4%)	0.292
Secondary endpoint							
Angina pectoris	9 (9.9%)	7 (9.5%)	2 (11.8%)	0.774	0 (0.0%)	9 (15.3%)	0.020*

*P < 0.05.

A post-LCX-QFR <0.8 was associated with a higher 1-year incidence of cardiovascular death or MI (*P* = 0.036). However, no statistically significant difference was observed in the primary endpoint compared with the QFR ≥0.8 group (*P* = 0.235). All nine patients with persistent angina pectoris were in the ΔQFR <0 group (*P* = 0.020).

ΔQFR proved to be a robust predictor of the 1-year incidence of the primary endpoint, with an incidence of 15.3% in the ΔQFR ≥0 group compared to 0% in the ΔQFR <0 group [area under the curve (AUC): 0.822; 95% CI: 0.728–0.895, *P* < 0.001]. The optimal cutoff value of ΔQFR for predicting adverse events was ≤−0.03. Using this cutoff, the Kaplan–Meier survival analysis showed that the ΔQFR ≤−0.03 group had a significantly increased risk of experiencing the 1-year primary endpoint compared to the ΔQFR >−0.03 group (*P* = 0.003). However, no statistically significant differences were observed in 1-year cardiovascular death (*P* = 0.248), target bifurcation-related MI (*P* = 0.248), revascularization in LM-LAD (*P* = 0.831), revascularization in LCX (*P* = 0.082), or revascularization in both arteries (*P* = 0.100) (Figure 3).

### 3.3 Predictors for low QFR

**Figure 5:**
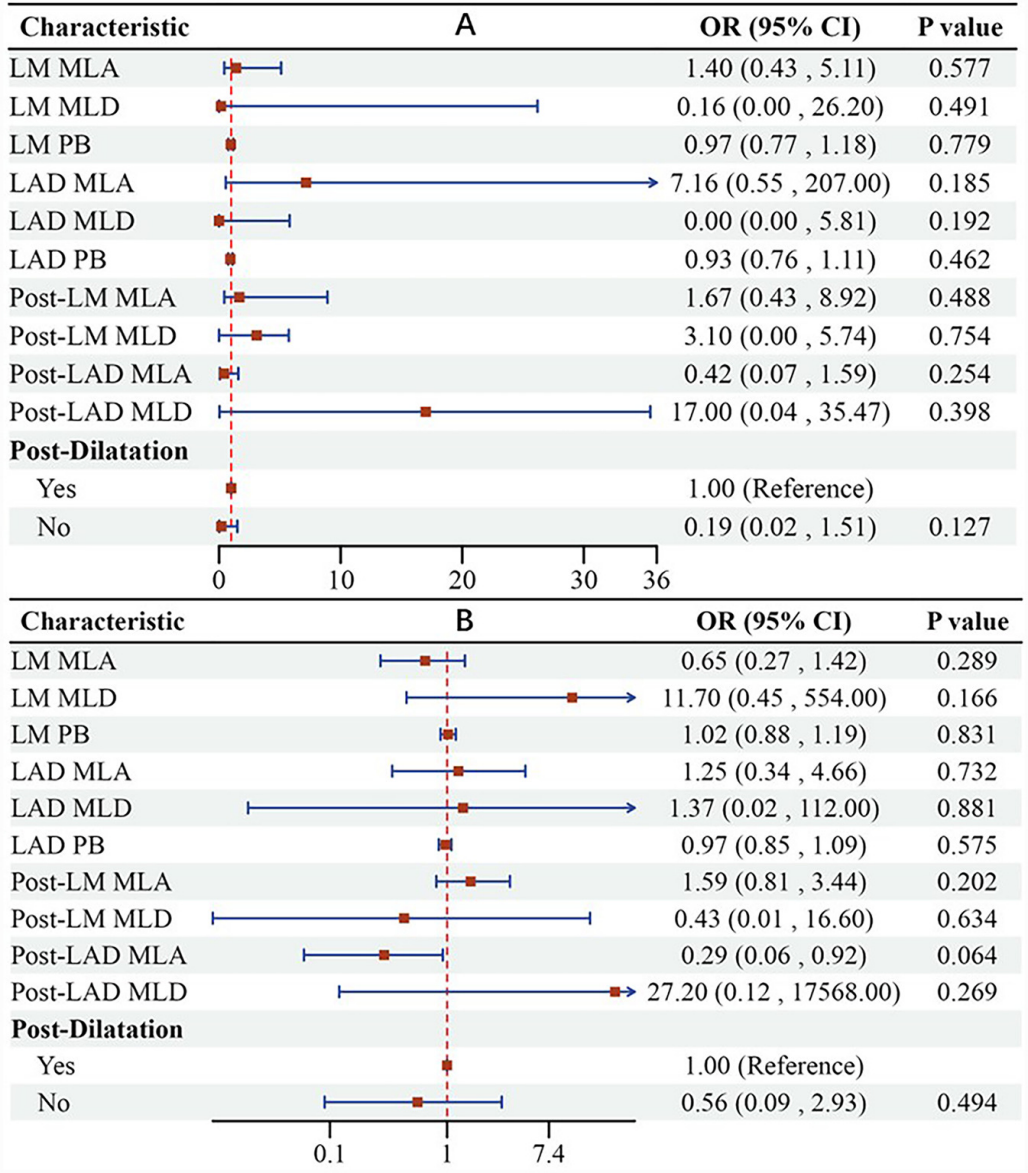
Forest plot suggesting that IVUS evaluation results of pre/post-LM-LAD are not significant independent predictors of postoperative QFR <0.8 **(A)** or ΔQFR ≤−0.03 **(B)**. LM, left main coronary artery; LAD, left anterior artery; MLA, minus lumen area; MLD, minus lumen diameter; PB, plaque burden.

The findings from the multivariate regression analysis were illustrated in a forest plot, indicating that the IVUS evaluation results of pre/post-LM-LAD are not significant independent predictors of a postoperative QFR <0.8 or ΔQFR ≤−0.03 (Figure 5). However, a larger post-LAD minus lumen area (MLA) appears to be likely related to a decline in QFR (*P* = 0.064).

## 4 Discussion

LM-CBLs are rarely focal. IVUS data demonstrate that, in over 90% of cases, plaques extend from the LM into the proximal LAD. Furthermore, in 66% of cases, these plaques reach the proximal LCX, and in 62% of cases, they involve both the LAD and LCX branches. The provisional single-stent strategy has emerged as a potentially effective approach for the interventional management of left main bifurcation lesions. Postoperative physiological assessments can elucidate the functional significance and clinical implications of left main bifurcation lesions, offering the potential to enhance clinical outcomes in such cases (8). Previous research has shown that the use of rewire guidewires, which are advanced through stent struts into lateral branches, can complicate the procedure and is associated with a failure rate nearing 10%. QFR may serve as a reliable alternative for postoperative hemodynamic evaluation in patients with left main bifurcation lesions. A QFR threshold of <0.80 has been associated with a higher risk of cardiovascular death following LM bifurcation stenting (9, 10). Therefore, we examined the connection between LCX-QFR and clinical results following single-stent placement for LM bifurcation lesions using IVUS guidance at our clinical center.

The major findings from the present study are as follows: (1) functional ischemia, as assessed by QFR (ΔQFR < 0), was observed in approximately 65% of patients; (2) the IVUS-based lumen evaluation data from pre/post-LM-LAD did not effectively predict LCX-QFR outcomes; (3) patients in the ΔQFR <0 group had a significantly increased risk of achieving the 1-year primary endpoint and persistent or recurrent angina pectoris, especially when ΔQFR ≤−0.03; and (4) a post-LCX-QFR <0.8 was associated with a higher 1-year incidence of cardiovascular death or MI.

In a limited, single-center study involving 43 patients, LM-LAD crossover stenting led to 42% of cases showing angiographic diameter stenosis greater than 50%, an average reduction of 26% in the minimal lumen area, and a 7% occurrence of positive FFR at the LCX ostium after stenting (9). Post-IVUS analysis revealed that the MLA of the LCX ostium decreased to ≤4.0 mm^2^ following (provisional) crossover stenting, indicating the potential benefit of a two-stent approach for distal LM bifurcation lesions. The reduced minimal lumen area, along with increased vessel eccentricity and stable plaque mass, suggests that carina shift is the primary cause of acute lumen loss in the LCX. Nevertheless, carina shift is a localized occurrence and is infrequently linked to functional flow issues in the LCX (5). In our study, only 14 patients in the single-stent crossover group underwent a pre-PCI IVUS evaluation of the LCX. Following their surgeries, all of these patients declined additional assessments and treatments, relying solely on angiographic signs of thrombolysis in myocardial infarction (TIMI) III flow in the LCX. However, our retrospective analysis revealed a notable decrease in QFR. This decrease in QFR may indicate issues with microvascular function, ostial LCX remodeling, or issues related to carina shift, prompting the surgeon to evaluate the necessity for additional vascularization. A study involving 83 patients highlighted a significant visual-functional mismatch following LM-LAD stenting. The presence of a positive FFR after stenting was associated with a notably increased rate of target lesion failure over a 5-year follow-up period, with 33.4% in the positive FFR group compared to 10.7% in the negative FFR group. Furthermore, some researchers reported a comparable MACE rate of 18.1% at 1 year in their randomized study guided by FFR and angiography. They noted a somewhat increased revascularization rate in both the main and side branch vessels for angiography-guided PCI (11). Consequently, in conjunction with our research findings, we propose that patients with compromised LCX function following a single-stent strategy for LM-LAD should be considered for balloon dilatation at the LCX ostium to enhance both blood flow and overall prognosis. These patients should also receive closer clinical follow-up, repeat functional assessment, and alternative revascularization strategies when necessary.

Furthermore, no statistically significant differences were observed in the rates of 1-year cardiovascular mortality, target vessel revascularization, or related myocardial infarction, which may be attributable to the limited sample size of our investigation.

## 5 Limitations

Our study has several limitations that must be considered. First, the retrospective and single-center nature of this analysis, which was conducted using images not originally intended for QFR analysis, presents inherent limitations. Second, the sample size of our study was relatively small, and the cumulative incidence of 1-year endpoints was low. Therefore, our findings should be confirmed in larger prospective studies in the future. In addition, to date, only a 1-year follow-up has been completed. Follow-up is planned to continue for up to 3 years to further assess long-term outcomes. In addition, we performed IVUS assessment of LCX only in a small proportion of patients before PCI, which resulted in an inability to provide anatomical parameters that influence functional status after LCX. Finally, this study adopted a cutoff value of 0.80 for QFR-guided ischemia, although the precise cutoff for QFR remains debated, which could have impacted the final outcomes.

## 6 Conclusion

Our research indicates that the LM-LAD single-stent strategy is associated with an increased incidence of decline in LCX-QFR. Furthermore, a ΔQFR value of ≤−0.03 is correlated with the occurrence of major adverse cardiac events within 1 year, while a post-QFR value of <0.8 is linked to a heightened risk of cardiogenic death and myocardial infarction over the same period. Future investigations may enhance our understanding of optimal surgical approaches for LM bifurcation lesions by comparing anatomical outcomes guided by postprocedural physiology and intraluminal imaging, ultimately aiming to improve clinical prognoses.

## Data Availability

The original contributions presented in this study are included in the article/Supplementary Material, further inquiries can be directed to the corresponding authors.
